# Host *ALDH2* deficiency aggravates acetaldehyde metabolism disturbance and gut microbiota dysbiosis in chronic alcohol exposure mice

**DOI:** 10.3389/fmicb.2025.1617673

**Published:** 2025-07-22

**Authors:** Xueqin Tan, Beiyi Wu, Xue Wen, Yunzhu Li, Xuewen Xu

**Affiliations:** ^1^Research Laboratory of Plastic and Burns Surgery, West China Hospital of Sichuan University, Chengdu, China; ^2^Department of Plastic and Burn Surgery, West China Hospital of Sichuan University, Chengdu, China

**Keywords:** chronic alcohol exposure, mitochondrial acetaldehyde dehydrogenase 2 (ALDH2), acetaldehyde, ileum microbiota, 16S rRNA gene sequencing

## Abstract

Alcohol is inextricably linked with intestinal microbiota as it was absorbed through gut. While mitochondrial aldehyde dehydrogenase 2 (ALDH2), as the major enzyme responsible for metabolizing toxic acetaldehyde to acetate, is important factor influencing alcohol metabolism. However, it is not yet known the relationship between *ALDH2* knockout (KO) and gut microbiota profiles in mice under chronic alcohol exposure. Therefore, this study aimed to investigate the effect of 5% v/v alcohol exposure on the gut microbiota of *ALDH2* knockout (KO-5%) and wild-type (WT-5%) mice. At the end of 10-week experiment, KO-5% mice exhibited a higher serum acetaldehyde concentration and upregulated expression of pro-inflammatory cytokines in intestine tissue than WT-5% mice. Metagenomic results revealed that the KO-5% mice had a significant decrease in alpha diversities. Moreover, KO-5% mice exhibited gut microbiota dysbiosis with the characteristic of a higher abundance of phylum *Proteobacteria*, and genera *Stenotrophomonas* and *Ralstonia*, whereas the level of genera *Lactobacillus*, unclassfied *Bacilli*, and *Turicibacter* were decreased. Additionally, genera *Candidatus Arthromitus* and *Ralstonia* were the most representatives in the KO-5% mice. Further, chronic alcohol exposure resulted in enriched expression of genes associated with bacterial metabolism and cellular processes in gut from WT mice. Taken together, our findings demonstrated a strong interaction between *ALDH2* and the gut microbiota to response to alcohol exposure.

## 1 Introduction

Alcohol is a unique addictive substance consumed worldwide, exhibiting complexing pharmacological qualities in the body (Piano et al., 2025). Harmful alcohol use, encompassing alcohol abuse and chronic exposure has profound adverse effects on the human body and is well documented to be associated with causing social harm and financial burden (Axley et al., 2019). Alcohol is absorbed throughout the entire gastrointestinal tract, with a small proportion of alcohol absorption occurs in the mouse and esophagus, ~20% is absorbed in the stomach, followed by ~70% absorbed from the small intestine (Moreau et al., 1997). The rate of absorption and metabolism was affected by a number of factors, such as host conditions, the concentration of alcohol, the speed of which the drink was consumed, and whether the alcohol are drunk on an empty stomach (Butts et al., 2023). Recently, the possibility has been raised that alcohol intake may also influence the gut microbiota compositions and functions (Engen et al., 2015).

Mammalian gastrointestinal tract harbors trillions of microbes including bacteria, protozoa, viruses, archaea, and fungi. This population of microbes displays a broad range of symbiotic interactions with the host (Mallott and Amato, 2021; Sidhu and van der Poorten, 2017). Moreover, studies have provided compelling evidence for the critical role of gut microbiota in chronic alcohol exposure, one study using 16S rRNA sequencing method found that a lower abundance of *Bacteroidetes* and a higher abundance of *Proteobacteria* of mucosa was observed in alcoholics (Mutlu et al., 2012). Another study showed a reduction from *Bacteroidetes* and an increase from *Proteobacteria* and *Fusobacteria* in human subjects with alcohol-related cirrhosis (Chen et al., 2011). In addition, it is now generally accepted that gut microbiota is one of the critical players in alcohol-induced injury in body, and the bacterial products appears to play a key role in the progression of alcohol associated liver disease (Vassallo et al., 2015; Way et al., 2022). Nevertheless, the etiology of alcohol-induced injury is a complex interplay of factors, and these factors determining alcohol toxicity remain unclear, implying that further studies are needed to clarify the interactions between gut microbiome and other factors (Wang et al., 2024).

After absorbed in the gastrointestinal tract, alcohol circulated in the blood and diffused into organs. Then, oxidative alcohol metabolism take place mainly in the liver, where most alcohol was oxidized to toxic acetaldehyde (Zakhari, 2006; Foster and Marriott, 2006). Three separate enzyme systems have been identified to catalyze oxidative alcohol metabolism, among them, mitochondrial aldehyde dehydrogenase 2 (ALDH2), as one of the major rate-limiting enzymes in this reaction, is mainly responsible for detoxifying toxic acetaldehyde to non-toxic acetic acid (Cederbaum, 2012). Excessive accumulation of acetaldehyde has cytotoxicity since acetaldehyde has the capacity to bind to proteins, form carcinogenic with DNA, and forms adducts with neurotransmitter, which can cause DNA even organ damage, resulting in a range of metabolic diseases (Park et al., 2016; Chen et al., 2019). Although the amount of ALDH2 in the liver is abundant, the activity that is closely connected with the *ALDH2* genotypes (Chen et al., 2022). Specifically, there is a significant genetic polymorphism (rs671, G>A) of the *ALDH2* gene, resulting in allelic variant ALDH2*1 (G) and allelic variant ALDH2*2 (A). Of note, the allelic variant ALDH2*2 has been identified to reduced ALDH2 enzymatic activity in comparison with ALDH2*1 (Edenberg, 2007). People with one or especially two copies of ALDH2*2 allele exhibited higher acetaldehyde levels after alcohol consumption. Moreover, the ALDH2*2 dysfunctional variant causes Asian flush syndrome or other uncomfortable symptoms during alcohol consumption in more than 560 million people worldwide, especially in east Asians (Wall et al., 2000; Chen et al., 2020). Previous works performed on mice revealed that *ALDH2* knockout or deficiency led to increased sensitivity to alcohol-related DNA damage and tissue injury (Kwon et al., 2014; Ma et al., 2010; Matsumoto et al., 2014; Rungratanawanich et al., 2023). Additionally, the host gene expression may affect intestinal microbial composition (Goodrich et al., 2017; Davenport, 2016). However, the crosstalk between *ALDH2* and the composition of gut microbiota in chronic alcohol exposure and the detailed mechanisms is still unclear. Therefore, exploring the relationship between *ALDH2* and gut microbiota in alcohol exposure is meaningful and worthwhile, and we are aimed to investigate the gut microflora composition and function in *ALDH2* KO and WT mice after exposure to alcohol in this study.

## 2 Materials and methods

### 2.1 Animals

All animal protocols were approved by the Animal Ethics Committee of West China Hospital of Sichuan University (Code: 20230228133). Six-week-old *ALDH2* knockout (KO) and wild-type (WT) mice on C57BL/6 background were bought from Cyagen Biosciences lnc. (Suzhou, China). KO individuals were generated by mating heterozygous mice as described previously (Qiu et al., 2023). All mice were housed in a SPF facility (five animals per cage) under a 12-h day and night lighting cycle with free access to food. To confirm the efficiency of our knockouts, the *ALDH2* expression at the mRNA and protein level in both liver and ileum tissues was measured by real time PCR and western blot analysis, respectively. The primer sequences of *ALDH2* and *18 s* were presented in Table 1. GAPDH and ALDH2 protein levels were detected by rabbit anti-GAPDH antibody (Cell Signaling Technology, 1:1000) and rabbit anti-ALDH2 antibody (Proteintech, 1:2000).

**Table 1:** Information of the primers used for qPCR reactions.

Gene	Forward sequence (5′–3′)	Reverse sequence (5′–3′)
*18 s*	TTGACTCAACACGGGAAACC	AGACAAATCGCTCAACCAAC
*ALDH2*	CCTGAGCCGAATGCTTTTAAG	CTCACGCTCCTTACTGGAC
*TNF-α*	CCCTCACACTCAGATCATCTTCT	GCTACGACGTGGGCTACAG
*IL-1β*	GCAACTGTTCCTGAACTCAACT	ATCTTTTGGGGTCCGTCAACT
*IL-6*	CCAAGAGGTGAGTGCTTCCC	CTGTTGTTCAGACTCTCTCCCT
*IL-10*	GCTCTTACTGACTGGCATGAG	CGCAGCTCTAGGAGCATGTG

### 2.2 Chronic alcohol exposure

WT and KO C57BL/6 male mice were used to construct chronic alcohol exposure model through drinking 5% concentration (v/v) of alcohol in their water. The alcohol exposure lasted for 10 weeks. Based on alcohol consumption and *ALDH2* genotype, mice were randomly divided into four groups: (1) KO mice were fed 5% alcohol (KO-5%, *n* = 5), (2) KO mice drinking water (KO-Control, *n* = 5), (3) WT mice receiving 5% alcohol (WT-5%, *n* = 5), and (4) WT mice receiving water (WT-Control, *n* = 5). The water intake (g) and body weight (g) were recorded weekly. At the end of the experiment, all mice were deeply anesthetized with sodium pentobarbital (50 mg/kg), and blood from the heart chambers were collected and stand at room temperature for 1 h, then centrifuged for 15 min at 3,500 × g at 4°C to separate serum. The level of serum ethanol was measured by Ethanol Assay Kit (BioVision; K620-1001, United States) according to manufacturer’s instructions. Concentrations of serum acetaldehyde were determined using aldehyde Quantitation Kit (AAT Bioquest; 10,051, United States). Meanwhile, intestinal contents were collected from approximately the middle region of ileum, and immediately frozen in liquid nitrogen and subsequently transferred to a −80°C freezer until DNA extraction.

### 2.3 RNA extraction and quantitative real-time PCR (qPCR)

Intestinal tissue samples were used to isolate RNA and differential expression of major inflammatory factors was detected through qPCR. Total RNA was extracted using Cell Total RNA Isolation Kit following manufacturer’s protocol (RE-03111, FOREGENE, China), and RNA concentrations and purities were measured by NanoDrop (Thermo Fisher Scientific Inc.). Subsequently, the first-strand cDNA was synthesized with an iScript cDNA Synthesis Kit (Bio-Rad, Hercules, CA, United States) according to the manufacturer’s recommendations. After RNA purification and cDNA synthesis, qPCR was performed with iTaq Universal SYBR Green Supermix (Bio-Rad, Hercules, CA, United States) on the CFX Connect Real-Time System with the cycling conditions: 95°C for 10 min; 40 cycles of 95°C for 10 s and 60°C for 40 s with fluorescent reading. Transcript levels were analyzed by comparative ΔΔCt method and *18 s* served as endogenous reference control. Specific primers sequences are listed in Table 1.

### 2.4 DNA isolation and 16S rDNA sequencing

The microbial DNA was isolated from approximately 0.4 g of intestinal content samples using TIANamp stool DNA isolation Kit (Qiagen, Shanghai, China) according to the manufacturer’s protocol. The concentration and integrity of the DNA was assessed using Nanodrop ND1000 spectrophotometer (Nanodrop Technologies, Mont-chanin, DE, United States) and 2% gel electrophoresis, respectively. Afterwards, 30 ng of DNA was used as template to amplify the V3-V4 region of the 16S rDNA by using the universal primers 338F (5′-ACTCCTACGGGAGGCAGCA-3′) and 806R (5′-GGAC-TACHVGGGTWTCTAAT-3′). Subsequently, the PCR products were checked by visualization with gel electrophoresis, and quantified for sequencing libraries construction. Finally, the qualified libraries were sequenced paired-end on the Illumina HiSeq 2,500 platform.

### 2.5 Bioinformatics and data analysis

The raw reads were initially processed using the Quantitative Insights into Microbial Ecology2 (QIIME2 v.2023.5) open-source bioinformatics tool (Bolyen et al., 2019). Pair end reads were joined and the Debular program (Amir et al., 2017) was used to demultiplexed sequences. Following this, sequences can be assigned into operational taxonomic units (OTUs), and the OTUs were taxonomically classified based on the Silva database (v.138) (Quast et al., 2013). Then, the alpha diversity metrics including Shannon and Simpson indices were evaluate by using the Kruskal-Wallis test. For beta diversity, calculation of the Bray Curtis distance and Unweighted Unifrac metrics were performed with the QIIME2 platform, and the ggplot2 (v3.5.0) package was used to visualize these metrics. The characterization of bacterial features was performed using the Linear discriminant analysis (LDA) effect size (LEfSe) for biomarker discovery (Segata et al., 2011). Finally, the software PICRUSt2 (v1.7.3) (Douglas et al., 2020) was enrolled to investigate if there were functional similarities between groups, and the resulting pathway abundance data was visualized using the ggpicrust2 package. Statistical analyses were performed using R (v4.3.2) software.^1^ Differences between pairs of means were considered significant when *p* < 0.05.

## 3 Results

### 3.1 Animal model establishment of chronic alcohol exposure

**Figure 1:**
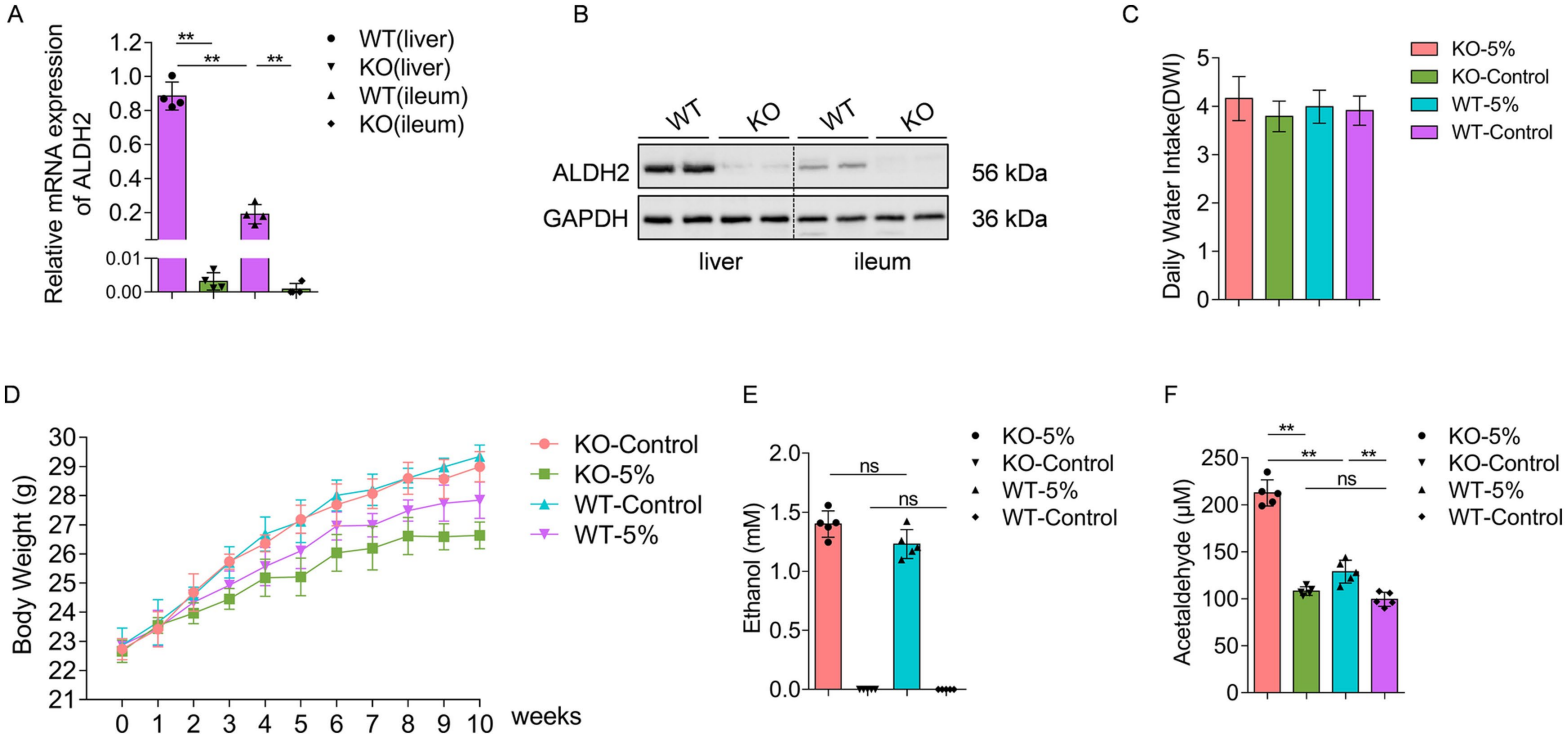
Establish a chronic alcohol exposure animal model. The level of ALDH2 at mRNA **(A)** and protein **(B)** was highly expressed in WT mice when compared with KO mice in both liver and ileum tissues. The daily water intake **(C)** and body weight **(D)** during the 10-week alcohol exposure had no significant differences across different experimental groups. Ethanol concentrations **(E)** and acetaldehyde concentrations **(F)** were measured at the end of experiment among different groups. Data are presented as “mean ± SD,” *p < 0.05, **p < 0.01.

In order to evaluate the *ALDH2* knockout efficiency of KO mice, we examined the *ALDH2* expression profiles in both liver and ileum tissues by real time PCR and western blot analysis. The results showed that *ALDH2* mRNA (Figure 1A) and protein (Figure 1B) were highly expressed in WT mouse liver and ileum tissues, whereas an undetectably low level in both tissues regarding KO mice. After 10-week ethanol exposure, the average daily water intake in mice of the four groups were similar, without significant difference between the WT and WT-5% groups, or between the KO and KO-5% groups (Figure 1C). Further, the body weight was recorded and we found that both KO-5% and WT-5% mice had a lower body weight gain when compared with KO-Control and WT-Control mice (Figure 1D). Additionally, ethanol clearance in acetaldehyde metabolism was analyzed, which showed that, although sera ethanol concentration did not show significant differences in the KO-5% compared to WT-5% mice (Figure 1E), the serum acetaldehyde level was remarkably elevated in the KO-5% mice than that in the WT-5% mice (*p* < 0.01) and KO-Control (*p* < 0.01) (Figure 1F). These findings suggested that the chronic alcohol exposure induced decreases in body weight gain, and the loss of *ALDH2* gene can inhibiting the elimination of blood acetaldehyde.

### 3.2 Chronic alcohol exposure exacerbated intestinal inflammation in *ALDH2* knockout mice

**Figure 2:**
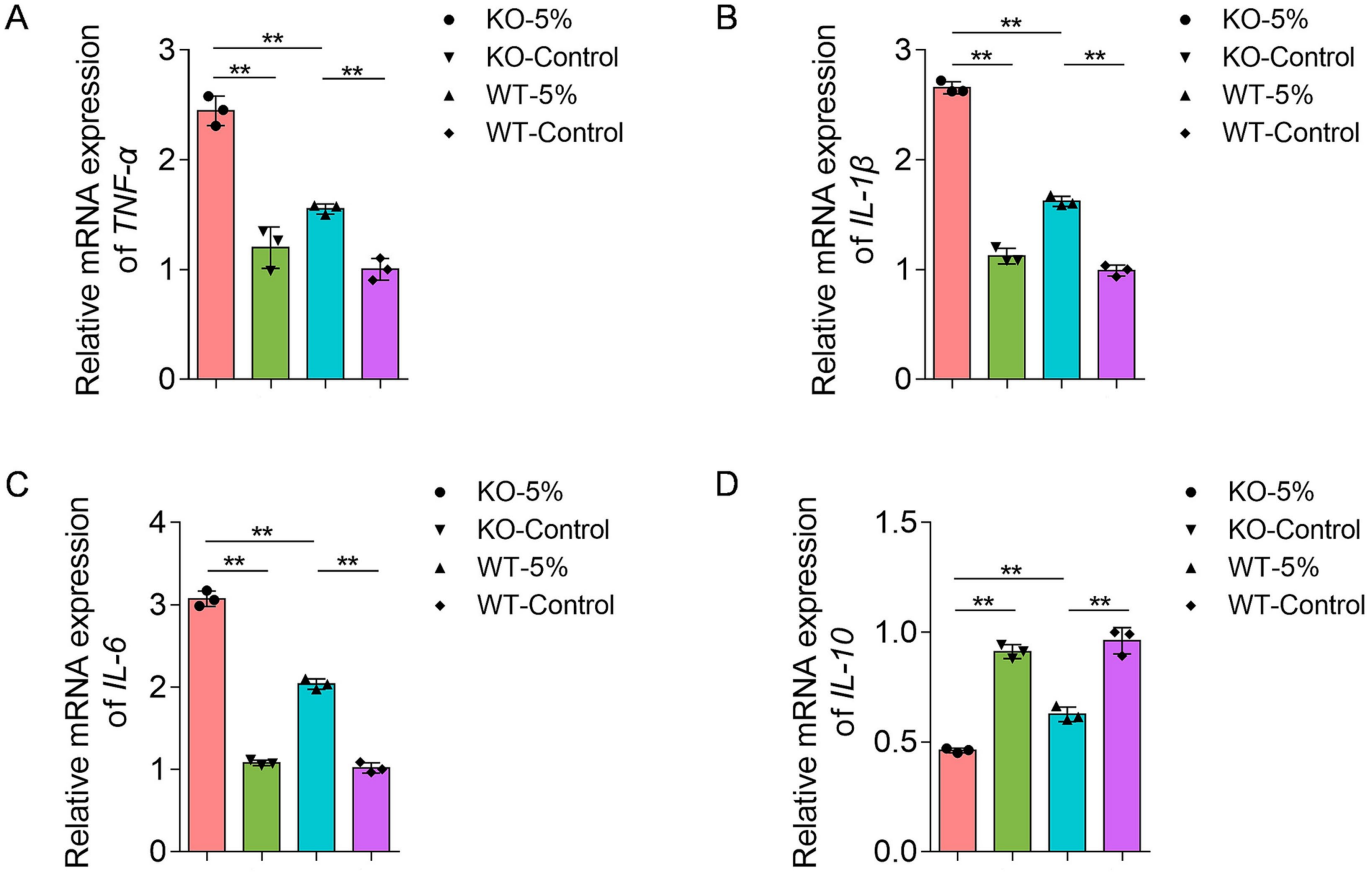
Effects of chronic alcohol exposure on the mRNA expression of inflammatory biomarkers in *ALDH2* knockout mice. **(A)** Expression of *TNF-α*. **(B)** Expression of *IL-1β*. **(C)** Expression of *IL-6*. **(D)** Expression of *IL-10*. Data are presented as mean ± SD (*n* = 3). *p < 0.05, **p < 0.01.

Differential mRNA expression of inflammatory cytokines, including *TNF-α*, *IL-1β*, *IL-6*, and *IL-10*, was analyzed in the four groups. After 10-week chronic alcohol exposure, the relative mRNA levels of *TNF-α*, *IL-1β*, and *IL-6* were significantly increased in KO-5% and WT-5% mice compared to KO-Control and WT-Control groups, respectively (Figures 2A–C). Moreover, KO-5% mice exhibited the most pronounced elevation in all pro-inflammatory cytokine levels relative to other groups. This cytokine profile demonstrated that ethanol-induced intestinal injury, with *ALDH2* deficiency exacerbating the inflammatory response. Meanwhile, *IL-10* exhibited opposite expression patterns across the four groups (Figure 2D), suggesting its potential role in suppressing excessive inflammation responses through negative feedback regulation.

### 3.3 Chronic alcohol exposure reduced microbial community diversity in *ALDH2* knockout mice

**Figure 3:**
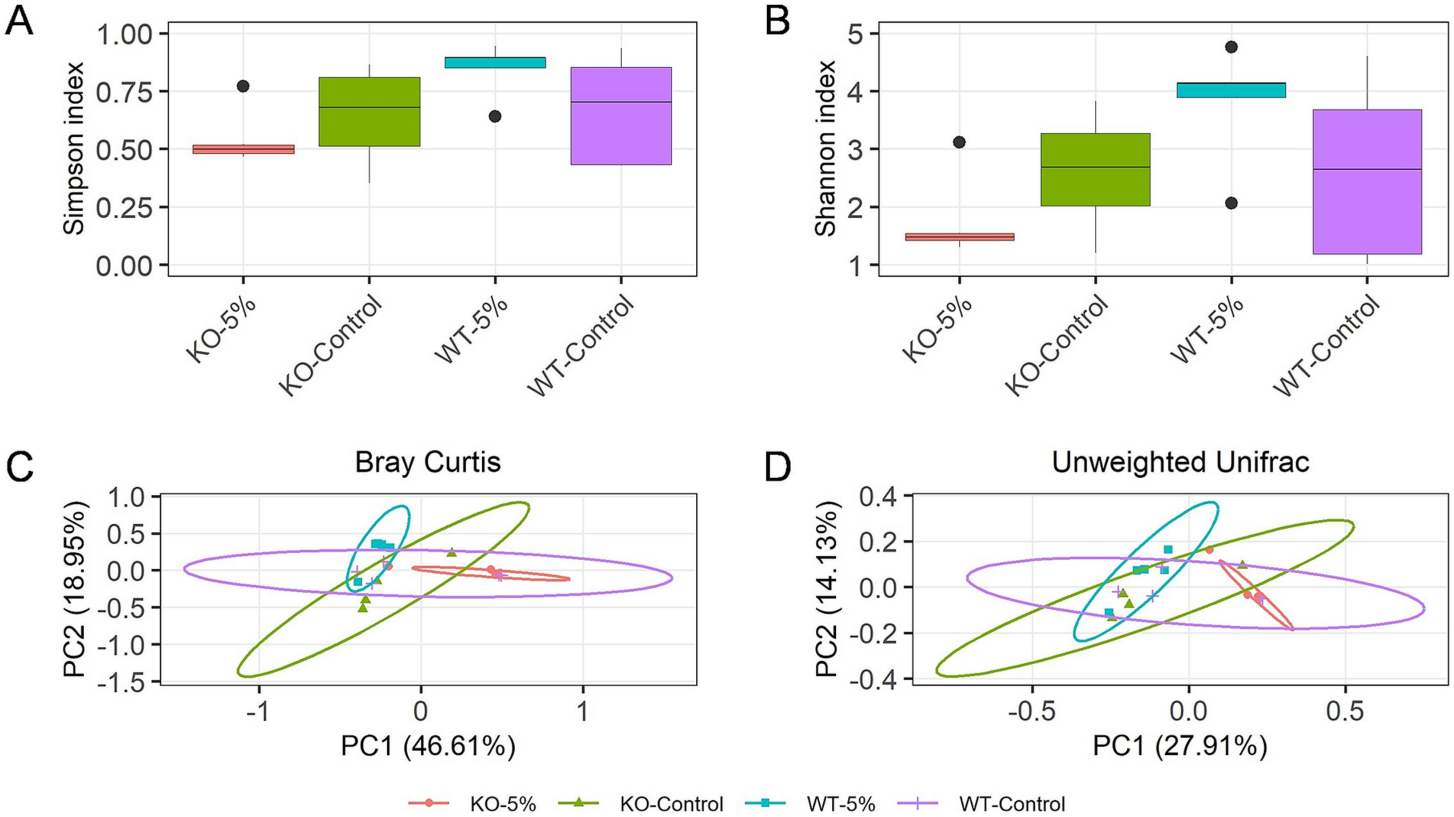
Chronic alcohol exposure resulted in distinct gut microbiota profiles in KO and WT mice. Alpha diversity within samples was measured by Chao1 **(A)** and Shannon index **(B)**. Scatterplot from Principal Coordinate Analysis (PCoA) of beta diversity was measured by Bray-Curtis index **(C)** and Unweighted Unifrac distance **(D)**. Results were determined by Kruskal–Wallis test when *p < 0.05, **p < 0.01.

Changes in gut microbiota diversity between the four groups of mice were analyzed. The Chao1 and Shannon indexes showed that the alpha diversities were significantly reduced in KO-5% when compared to the WT-5% mice (*p* = 0.028 for Chao1 richness index, Figure 3A; *p* = 0.011 for Shannon diversity index, Figure 3B). Moreover, the principal-coordinate analysis (PCoA) results based on both Bray Curtis (Figure 3C) and Unweighted Unifrac distances (Figure 3D) were used to measure the beta diversity in each group. The results showed that a notable shift in beta diversities was observed as the bacterial members and structure of the KO-5% mice were different from that of the WT-5% mice. Therefore, it was concluded that the *ALDH2* loss might reduce the richness and diversity of intestinal bacterial species when mice were under chronic alcohol exposure.

### 3.4 Chronic alcohol exposure changed gut bacterial composition in *ALDH2* knockout mice

**Figure 4:**
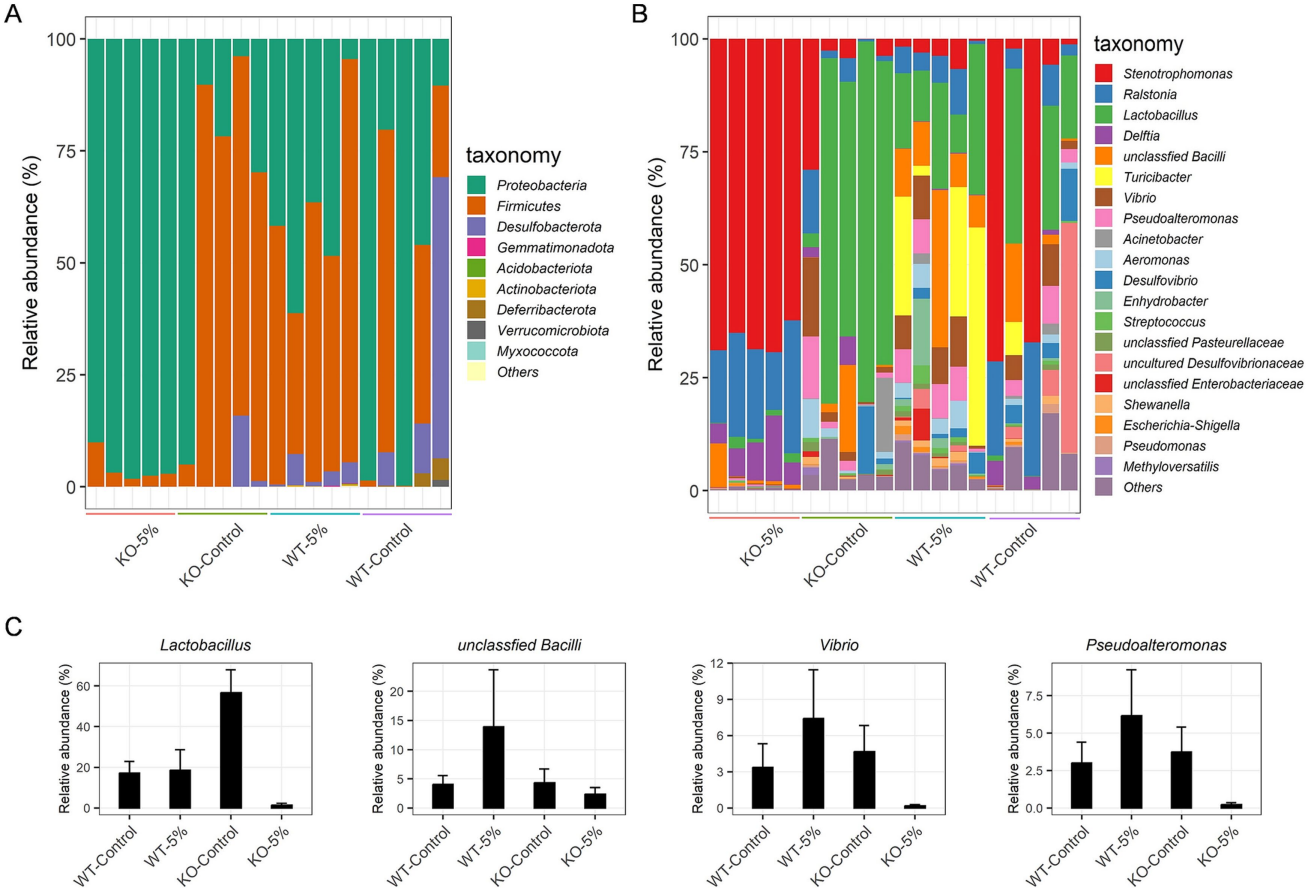
Chronic alcohol exposure resulted in distinct community structure in KO and WT mice. Summary of the relative abundances of bacterial phyla **(A)** and the top 20 most abundant bacterial genera **(B)**. Each color represents one bacterial species on the stacked bar chart. Relative abundance of special bacteria genera that expressed higher in WT-5% than WT-Control but which has lower abundance in KO-5% than KO-Control mice **(C)**.

We focused on the bacterial taxa relative abundance at the phylum and genus level. Among these phyla, *Proteobacteria* (95.97% ± 3.31) was the predominant phylum in KO-5% mice, but a significant decreased abundance of *Proteobacteria* was observed in KO-Control, WT-5%, and WT-Control animals with 16.38, 57.97, and 26.78%, respectively (Figure 4A). Moreover, in KO-Control and WT-5% mice, the predominant phylum was *Firmicutes* (79.33 and 57.97%, respectively), whereas that was decreased sharply in KO-5% mice. At the genus level, the most abundant taxa were *Stenotrophomonas* in KO-5% mice, followed by *Ralstonia*, *Delftia*, unclassfied *Bacilli* and *Lactobacillus* (Figure 4B). Contrarily, the WT-5% inhibited the growth of these taxa. Of note, *Lactobacillus*, unclassfied *Bacilli*, *Vibrio*, and *Pseudoalteromonas* were increased in WT-5% compared to WT-Control mice but they were less abundant in KO-5% compared to KO-Control mice (Figure 4C).

**Figure 5:**
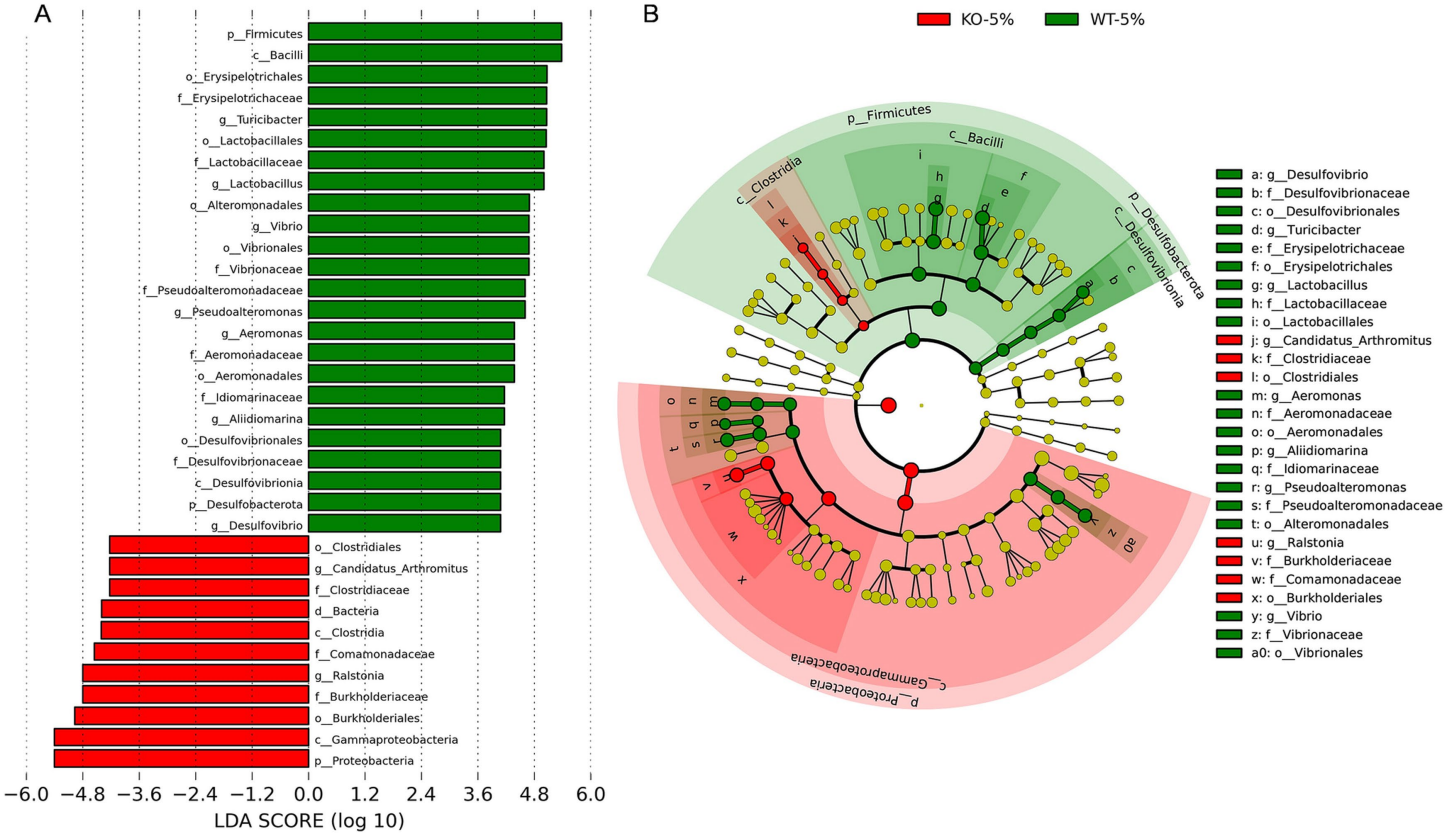
Differential represent taxa between KO-5% and WT-5% mice on the basis of Linear discriminant analysis effect size (LEfSe) analysis. Histogram of Linear Discriminant Analysis (LDA) scores indicating statistical different relative levels across all taxa **(A)**. Cladogram revealing the phylogenetic relationships between taxa with the statistically different **(B)**. Circle sizes in the cladogram plot represent the proportion to bacterial abundance, and the yellow circles represent different taxon with no obvious difference.

As a complementary and validating method to identify specific differences in taxa, the LEfSe analysis was performed between WT-5% and KO-5% mice with a threshold of LDA values > 4. Firstly, we generated a bar plot of the effect size of taxa and found 7 significantly enriched genera in the WT-5% mice, including *Turicibacter*, *Lactobacillus*, *Vibrio*, *Pseudoalteromonas*, *Aeromonas*, *Alidiomarina*, and *Desulfovibrio*. Whereas genera *Candidatus Arthromitus* and *Ralstonia* were the most representatives in the KO-5% mice (Figure 5A). Of note, all genera except for *Alidiomarina*, and *Candidatus Arthromitus* were presented in the top 20 abundant taxa. Secondly, we performed a cladogram to show the relationship between different taxa (Figure 5B). This figure illustrated several taxonomic groups such as *Lactobacillus* and *Turicibacter* within *Fimicutes* phylum, in particular the class *Bacilli*, were at a significant higher abundance in WT-5% mice. Furthermore, genus *Ralstonia* was found to be a significantly high abundant taxa in KO-5% mice (Figure 5B).

### 3.5 Predicted functional differences of the gut microbiota between KO-5% and WT-5% mice

**Figure 6:**
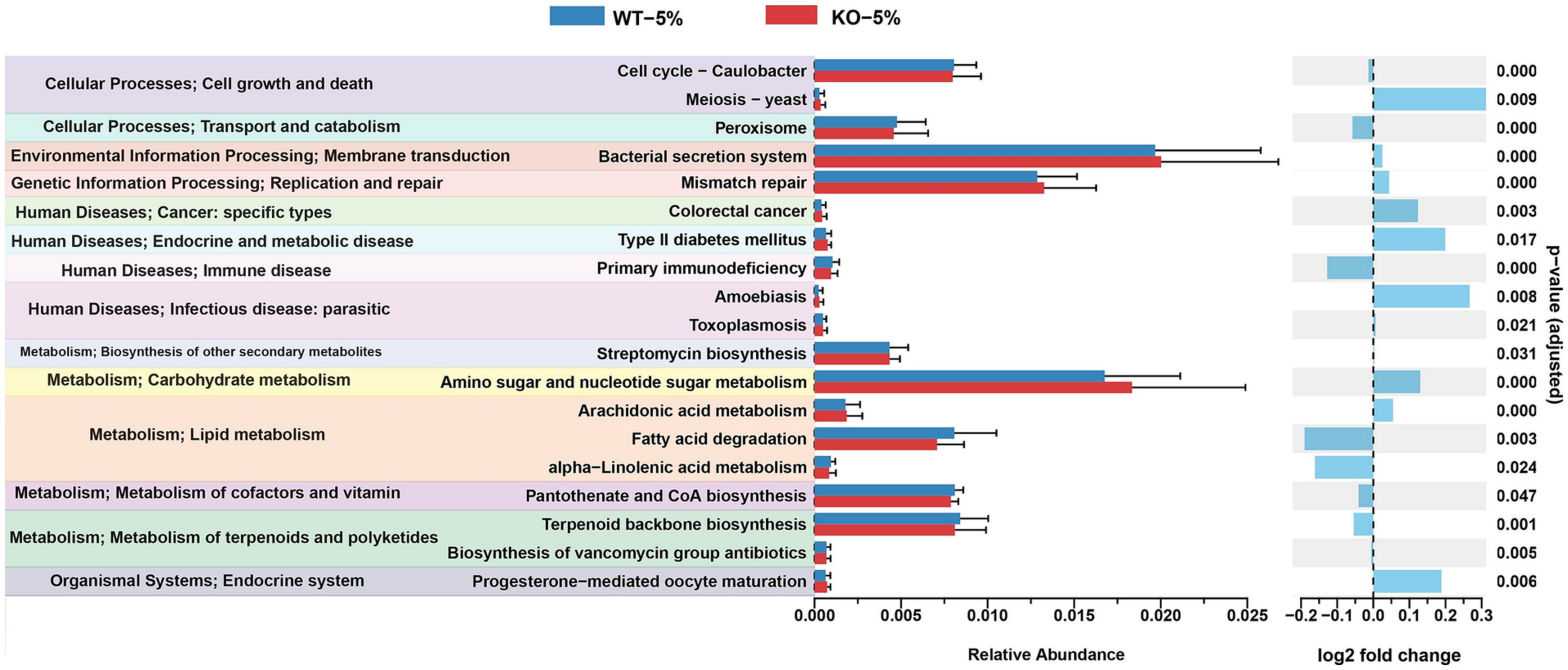
Differential abundances of bacterial functional pathways between KO-5% and WT-5% mice. All KEGG orthologs shown have an adjusted q value cutoff of 0.05.

Using PICRUSt2 software and KEGG database, we compared the gene abundance of the microbiota and inferred the profile of putative microbial functions. As illustrated in Figure 6, these genes were merged into level 3 KEGG orthologs, based on their cellular processes, environmental information processing, genetic information processing, human disease, metabolism, organismal systems. Notably, we found that the gut microbiota of KO-5% mice had relatively less bacterial containing genes for several pathways, such as peroxisome, primary immunodeficiency, fatty acid degradation, alpha-linolenic acid metabolism, pantothenate and CoA biosynthesis, terpenoid backbone biosynthesis, etc. The majority of these pathways were linked to aspects of bacterial metabolism and cellular processes, suggesting that the KO-5% showed a decreased metabolic response on the gut microbiota compared to WT-5% mice.

## 4 Discussion

The harms associated with alcohol are well documented, and heavy and long-term alcohol consumption were associated with serious physical and psychiatric illnesses such as liver disease, pancreatitis, depression, and dementia (Shimizu et al., 2016). Although various alcohol consumption patterns have been defined and the exact definitions are blurred, to date, acute and chronic drinking are the most relevant two alcohol drinking patterns for studying alcohol influence (Alcohol Research: Current Reviews Editorial Staff, 2018). In fact, the effects of acute and chronic alcohol consumption have been widely studied, most works have focused on alcohol-related liver disease because liver is the main site of alcohol metabolism (Massey and Arteel, 2012; Abbas et al., 2023). Additionally, recent works unveiled that alcohol intake impairs the function and homeostasis of gut microbiota (Wang et al., 2024; Leclercq et al., 2020), and further extended the interactions across host gene and gut microbiota in alcohol exposure murine model (Rungratanawanich et al., 2023; Liu et al., 2022).

It is well established that *ALDH2* gene mutation as well as *ALDH2* knockout induced an increased sensitivity to tissue injury under alcohol exposure (Chen et al., 2020; Ma et al., 2010; Matsumoto et al., 2014; Chen et al., 2014; Isse et al., 2005). In this study, we found that the *ALDH2* knockout contributes to higher sera acetaldehyde accumulation after exposure to 5% alcohol (Figure 1B), although they showed the same sera ethanol level, similar results were observed in mice that the loss of *ALDH2* leads to higher concentration of acetaldehyde in blood and multiple organs (Isse et al., 2005). These data indicated that *ALDH2* knockout causes a significant decrease in acetaldehyde scavenging capacity, resulting in evaluation of acetaldehyde level after drinking of alcohol (Chen et al., 2009). Although most alcohol is metabolized and converts into acetaldehyde in hepatocytes, the intestinal mucosa also expressed enzymes and contributed to oxidative of metabolism of alcohol (Chen et al., 2015). Besides, evidence has showed that alcohol is not directly metabolized by gut microbiota (Martino et al., 2022), but alcohol exposure can alter gut environment and microbiota structure and function. The significantly reduced Shannon indexes in KO-5% when compared to the WT-5% mice found in the current study are consistent with previous study performed on binge-on-chronic alcohol consumption mice (Shen et al., 2024), and heavy drinkers (Gurwara et al., 2020). In addition, the microbial community composition of KO-5% mice had the most significant dissimilarity from other groups. Therefore, this current study revealed that the alcohol exposure altered gut microbiota biodiversity in *ALDH2* knockout mice.

The KO-5% mice had the highest relative abundance of phylum *Proteobacteria*, while *Firmicutes* decreased significantly. Prior studies have found that alcohol drinking altered gut microbiota structure, with most of the data focusing on *Firmicutes* or *Bacteroidetes*, although the results were inconsistent (Lee et al., 2020; Peterson et al., 2017). *Proteobacteria* seem to have important role in inflammatory active and they were found with higher abundance in alcohol overconsumers. Moreover, many members of phylum *Proteobacteria*, including *Escherichia* have been associated with inflammation (Janket et al., 2020). In this study, the abundance of *Ralastonia* and *Stenotrophomonas* were obviously higher in KO-5% mice, suggesting that the inflammatory microbiota of *ALDH2* knockout mice may be increased. The genus *Lactobacillus* belongs to phylum *Firmicutes*, is a well-known probiotic with multiple health-promoting effects. Jung and colleagues (Jung et al., 2021) revealed that the *Lactobacilli* species as the indigenous flora had a potential to downregulate the alcohol and acetaldehyde levels, and whose effects depend on host *ALDH2* genotype. Increased abundance of *Lactobacilli* can alleviate the ill effects of alcohol consumption (Kim et al., 2022). Notably, a significantly reduced *Lactobacilli* was observed in *ALDH2* knockout mice, which perturbed intestinal bile acids metabolism and linked to sera proinflammatory cytokines such as *IL-1β*, *IL-6*, *TNF-α*, and C-reactive protein (Li et al., 2023). In line with these findings, we found the significantly decreased abundance of *Lactobacilli* as well as higher mRNA levels of *TNF-α*, *IL-1β*, and *IL-6* in KO-5% mice when compared to WT-5% individuals, which suggested that *Lactobacilli* played critical role in the *ALDH2* deficiency mice through the crosstalk with proinflammatory mediators. Based on prediction of functional pathways for gut microbiota, we found that several pathways, such as peroxisome, primary immunodeficiency, fatty acid degradation, alpha-linolenic acid metabolism, pantothenate and CoA biosynthesis, were more enriched in WT-5% mice. Of which, peroxisomes were closely related to lipid metabolism and reactive oxygen species turnover, and which were also required to innate immunity pathways (Cara et al., 2017). Furthermore, alpha-linolenic acid metabolism has been proven to alleviate inflammatory by restraining the NF-κB signaling pathway (Yang et al., 2023). These results suggested that the *ALDH2* played critical roles on the metabolic homeostasis.

Limitations of the current study are manifold. In our model setting we tested the sera ethanol concentration, serum acetaldehyde level, inflammatory cytokines, as well as intestinal flora at the end of the chronic alcohol exposure. This failed to present the systemic physiological changes over time, especially for the temporal dynamics of microbial communities. Furthermore, all mice enrolled in the study were selected based on their gender, age, body size and weight before our model setting, without prior characterization of the gut microbiota, which may be the causality that WT-Control mice had a relative diverse microbiome composition. Additionally, the intestinal barrier proteins such as ZO-1, Occludin, and Claudin are proven to be corrected with the dysbiosis of the intestinal microbiome (McMahan et al., 2023). It is critical to collected the corresponding tissues to determine whether gut microbiome dysbiosis corresponds to morphological damage.

## 5 Conclusion

This study demonstrated the effect of *ALDH2* on alcohol mediated gut microbiota dysbiosis upon chronic exposure to 5% alcohol. Host *ALDH2* knockout resulted in intestinal inflammation as well as gut dysbiosis, especially the increase of *Proteobacteria*, *Stenotrophomonas*, and *Ralstonia* proportion, while decrease of *Firmicutes*, *Lactobacillus*, unclassfied *Bacilli*, *Vibrio*, and *Pseudoalteromonas* proportion. Based on these findings, intestinal microbiota is closely connected to host *ALDH2* gene upon chronic alcohol exposure.

## Data Availability

The datasets presented in this study can be found in online repositories. The names of the repository/repositories and accession number(s) can be found at: https://www.ncbi.nlm.nih.gov/, PRJNA1245522.

## Footnotes

^1^ https://www.r-project.org/
